# Antibody reactivity to Afmp1p antigen in penguins with probable and confirmed aspergillosis

**DOI:** 10.3389/fvets.2025.1643473

**Published:** 2025-08-29

**Authors:** Adriana Callico, Mary Irene Thurber, Ellen Bronson, Julie Barnes, Anne Burgdorf-Moisuk, Nicholas A. Buscaglia, Michelle Davis, Ann Duncan, Ronan Eustace, Zoltan S. Gyimesi, Michael W. Hyatt, Sara Neumann, Colleen Clabbers, Fabiano Montiani-Ferreira, Carolyn Cray

**Affiliations:** ^1^Division of Comparative Pathology, Department of Pathology and Laboratory Medicine, Miller School of Medicine, University of Miami, Miami, FL, United States; ^2^Henry Vilas Zoo, Madison, WI, United States; ^3^Maryland Zoo in Baltimore, Baltimore, MD, United States; ^4^Santa Barbara Zoo, Santa Barbara, CA, United States; ^5^Dallas Zoo, Dallas, TX, United States; ^6^Sedgwick County Zoo, Wichita, KS, United States; ^7^Wildlife Conservation Society, New York Aquarium, Brooklyn, NY, United States; ^8^Detroit Zoological Society, Royal Oak, MI, United States; ^9^Potter Park Zoo, Lansing, MI, United States; ^10^Louisville Zoological Garden, Louisville, KY, United States; ^11^Busch Gardens Tampa Bay, Tampa Bay, FL, United States; ^12^Department of Veterinary Medicine, Universidade Federal do Paraná, Curitiba, Brazil

**Keywords:** antibody, aspergillosis, fungal disease, serology, penguin

## Abstract

Penguins under managed care are widely considered to have high susceptibility to infection by *Aspergillus* spp. Antemortem laboratory diagnostic options vary in sensitivity and specificity, and antibody detection has been problematic in penguin species given elevated levels of reactivity observed in clinically normal patients using traditional whole antigen enzyme-linked immunosorbent assays (ELISA). In the present study, an alternative assay was implemented to detect reactivity to Afmp1p, an *Aspergillus* cell wall antigen, in samples obtained from several different penguin species. With confirmed infection, abnormal protein electrophoretograms were consistently observed, and gliotoxin was detected in the majority of cases. An increase in reactivity to Afmp1p was observed in penguins with confirmed (*n* = 18, *p* < 0.0001) and probable (*n* = 13, *p* = 0.08) aspergillosis versus normal adult penguins (*n* = 33). Interestingly, increased reactivity to Afmp1p (p < 0.0001) was noted in normal adult penguins (*n* = 33) versus juvenile penguins (*n* = 22, *p* < 0.0001). Overall, the area under the curve for this assay was 0.890, with a sensitivity of 94.4% and a specificity of 57.6%, with an antibody assay cutoff of 1.0. Increasing reactivity resulted in an increase in specificity. These data support the use of Afmp1p antibody quantitation as part of a diagnostic workup in penguins with suspected aspergillosis.

## 1 Introduction

Aspergillosis remains a cause of morbidity and mortality in avian species, especially in penguins (1–3). While diagnostic options include complete blood count (CBC), fungal cultures, radiological and advanced imaging, and endoscopy, the availability of a sensitive and specific blood test would be preferred. However, antemortem laboratory diagnostics for avian aspergillosis remain challenging, with options ranging from antigen and antibody assays, gliotoxin detection, and plasma protein electrophoresis (1–5). An enzyme-linked immunosorbent assay (ELISA) using unfractionated whole *Aspergillus* antigen has been described in avian species, but its utility was limited as many birds have reactivity even with clinically normal status (6, 7). In penguins with confirmed infection, no significant difference was reported using this antibody assay; thus, it was not helpful as a diagnostic test (4). This lack of specificity led to studies of several other diagnostic assays, including the detection of circulating galactomannan, a major antigen of *Aspergillus* spp., and gliotoxin, a major toxin of *Aspergillus fumigatus* (8–10). In addition, protein electrophoresis has been described as a proxy test for aspergillosis through the demonstration of specific changes in globulin fractions, which are consistent with infection (4, 7). Finally, in penguins, hydroxybutyrate has also been proposed to be a sensitive biomarker of infection (4).

In falcons, a novel ELISA utilizing the Afmp1p antigen was described as a diagnostic tool (11). This secretory cell wall antigen has been described as a target antigen in the antibody response in humans (12). Based on a large sample set of normal falcons and a small sample set from those with confirmed aspergillosis, the specificity was reported as 99.2% and the sensitivity as 75%. The goal of the present study was to validate this novel assay as a diagnostic tool for aspergillosis in penguin. In addition, where sample volume permitted, antibody reactivity was examined in tandem with the panel of other laboratory diagnostic tests for aspergillosis.

## 2 Materials and methods

### 2.1 Samples

Samples were obtained in two modes. First, lithium heparinized plasma samples from both healthy and symptomatic penguins were submitted to the diagnostic laboratory (Avian & Wildlife Laboratory, University of Miami, Miami, FL, 33136, USA) as part of the panels inclusive of the tests that are presented in this study. These samples were shipped overnight in cold packs from the submitting institutions and analyzed on receipt. Second, frozen banked samples from various institutions were sent to the laboratory as part of a call for samples for this research study. Most of these latter samples were analyzed upon receipt, except for the samples from juvenile penguins, which were obtained as part of a planned avian malaria screening program and stored at -80°C until analysis.

The clinical groups are as follows: (1) normal juvenile African penguins (*Spheniscus demersus*, *n* = 22) were collected between July and October of 2021 from a single facility, which included 13 males and 9 females, with a median age 1.7 years (95% confidence interval (CI) 1.0–1.9); (2) normal adult African penguins (*n* = 33) were collected in April 2024 from a single facility, which included 17 males and 16 females, with a median age of 9.0 years (95% CI 6.4–12.0); (3) confirmed cases (*n* = 18) were collected between 2020 and 2024, which included 8 males and 10 females, with a median age of 9 years (95% CI 4.4–12.6). This group comprised 3 African penguins, 8 Humboldt penguins (*Spheniscus humboldti*), 1 gentoo penguin (*Pygoscelis papua*), 3 Magellanic penguins (*Spheniscus magellanicus*), and 3 little blue penguins (*Eudyptula minor*); and (4) probable cases (*n* = 13) were collected between 2020 and 2024, which included 6 males and 7 females, with a median age of 10 years (95% CI 3.6–12.5). The group comprised 10 African penguins, 2 Humboldt penguins, and 1 Magellanic penguin. Confirmed cases were supported by clinical signs; hematologic and other clinical pathology changes; radiographic and/or imaging evidence, which were consistent with fungal infection; and postmortem histopathologic diagnosis. Probable cases were classified by clinical presentation, clinical pathology findings, radiographic/imaging evidence, and positive response to anti-fungal treatment.

### 2.2 Afmp1p antibody assay

Recombinant Afmp1p was produced by Biomatik USA, LLC (Wilmington, Delaware, 19,809, USA) as previously described (11). Briefly, the N-terminal signal peptide sequence was removed, and a His-tag was added. Protein expression was performed in *E. coli*. Soluble protein was obtained by affinity purification and subsequently lyophilized after extensive dialysis against PBS (pH 7.4). Lyophilized protein was reconstituted using sterile water and stored as aliquots at –80°C. An indirect ELISA was used with Afmp1p antigen at a concentration of 1 μg/mL. Samples were diluted 1:8 in phosphate-buffered saline, and 50 μL of the sample was tested in duplicate. After an initial 30-min incubation at 37°C, the ELISA plate was washed three times, and then, 50 μL of a 1:600 dilution of horseradish peroxidase (HRP)-conjugated rabbit anti-chicken IgY—known to be reactive with penguin antibody (Millipore Sigma, Milwaukee, WI, 53209 USA)—was used for an additional 30-min incubation. After washing three times, 50 μL of 2,2′-azino-di(3-ethylbenzthiazoline-6-sulfonate) (ABTS, SeraCare, Milford, MA, 01757 USA) was added for a brief incubation at room temperature. Absorbance was then read at 405 nm using a FLUOstar Omega reader (BMG LABTECH, Cary, NC, 27513, USA). The results are expressed in optical density units.

### 2.3 Protein electrophoresis

Plasma protein electrophoresis was conducted using Split Beta Gels on the SPIFE 3000 system (Helena Laboratories, Beaumont, TX 77705, USA), following the manufacturer’s instructions, as previously described for penguin species (4).

### 2.4 Gliotoxin detection

Plasma samples were analyzed for the presence of gliotoxin using an Agilent Technologies 1,260 LC coupled to a 6,460 triple quadrupole MS (Agilent Technologies, Santa Clara, CA 95051, USA) as previously described (10). The limit of detection was 3 ng/mL. In the case study presentations, some data are presented as >3 ng/mL as the assay results were originally analyzed and reported as positive or negative based on this minimum detection level. More recent cases have been presented with quantitative results.

### 2.5 Statistical analysis

Basic descriptive statistics were conducted using Prism 6 (version 6.07, GraphPad Software, Boston, MA 02110, USA). Normality was assessed by the D’Agostino-Pearson test. Normal and non-normal data were determined so that the mean and standard error as well as the median and interquartile range could be presented. The Mann–Whitney U-test was conducted to determine differences when two study group comparisons were performed. Since the data did not meet the assumptions of normality, a Kruskal–Wallis test was also performed, making multiple comparisons among the four study groups. When a significant difference was detected, *post-hoc* pairwise comparisons were performed using Dunn’s test with Bonferroni correction to control for multiple testing. Spearman’s correlation analysis was used in the age analysis given the variable data distribution. The receiving operating characteristic (ROC) curve analysis with sensitivity and specificity was performed using MedCalc 23.1.7 (MedCalc Software Ltd., Ostend, Belgium) and Python (v3.11) with the libraries pandas, Matplotlib, and Pillow. Given the median age of the confirmed and probable groups, the normal adult group was utilized as the negative/normal group in these analyses.

## 3 Results

### 3.1 Comparison of study groups

A significant difference in antibody reactivity was observed with normal juvenile penguins (mean ± standard error (SE): 0.63 ± 0.05) having lower Afmp1p antibody reactivity compared to adult penguins (mean±SE: 1.01 ± 0.07, *p* < 0.0001). A moderate and significant correlation was observed between antibody reactivity in the normal adult penguin group and age (*r* = 0.45, *p* = 0.0088).

**Figure 1:**
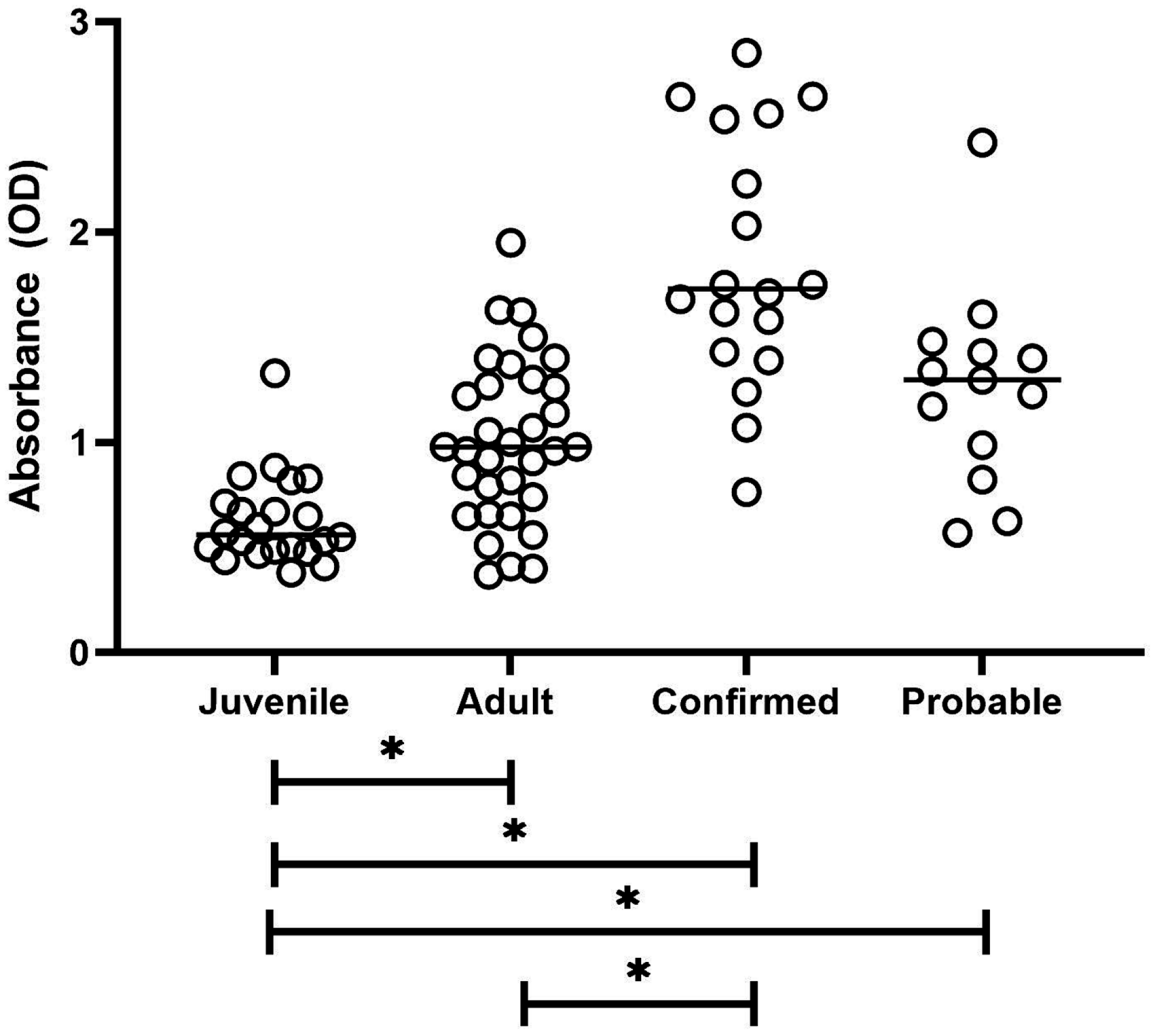
Plot of antibody reactivity by study group. Median group reactivity indicated by horizontal bar. Statistical significance for all group comparisons indicated by an asterisk is a *p*-value of <0.0001.

Differences between the study groups are shown in Figure 1. The Kruskal–Wallis test revealed a statistically significant difference in measurement values among the four study groups (*H* = 43.91, *p* < 0.0001). Samples from penguins with confirmed aspergillosis showed significantly higher Afmp1p reactivity than either clinically normal group (*p* < 0.0001). Note that the reactivity of the normal adult group was not significantly different from the probable group (*p* = 0.08).

**Figure 2:**
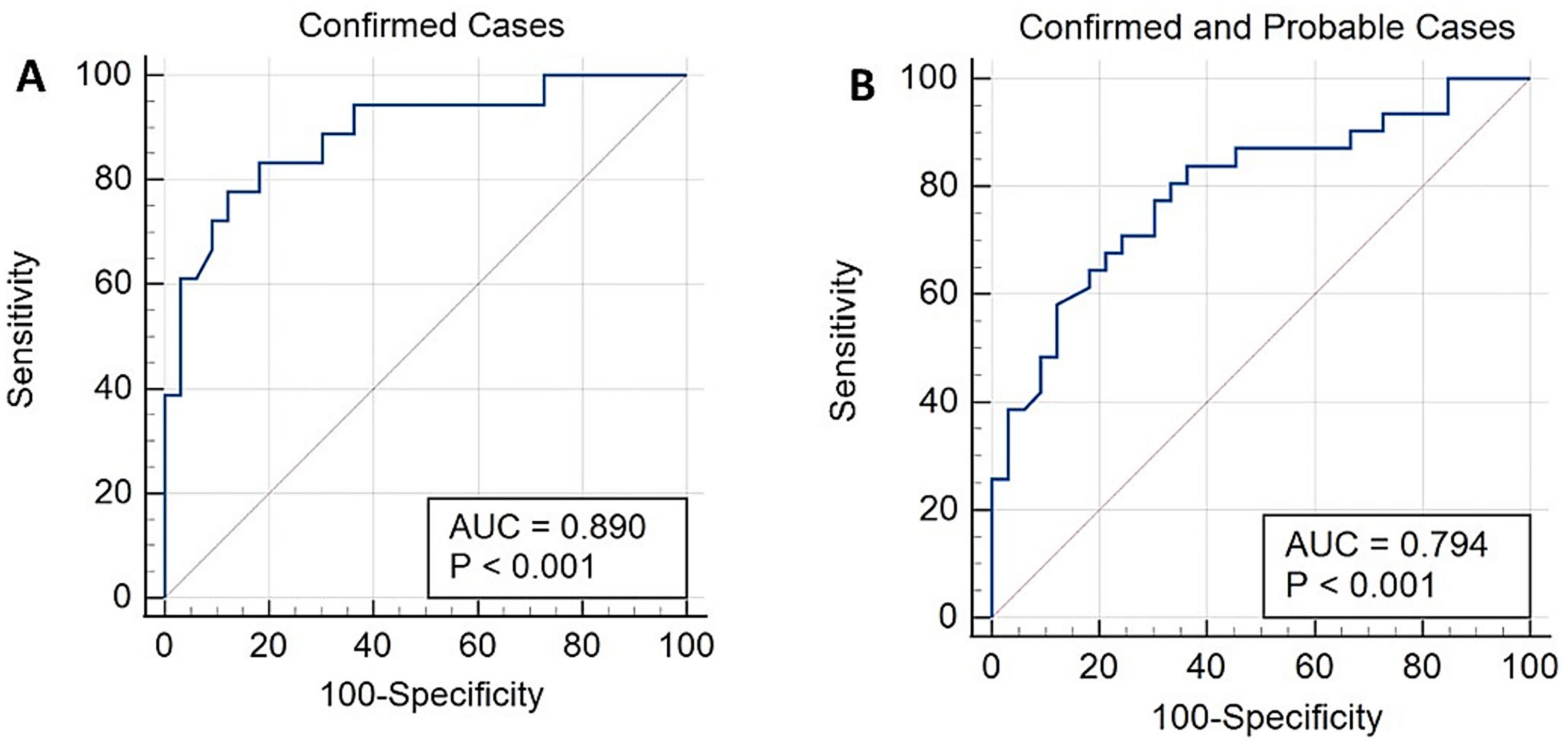
Receiver operating characteristic (ROC) curves, including only data from the confirmed study group **(A)** or combined data from the confirmed and probable groups **(B)**.

ROC curve analysis, which was limited to the normal adult group and the confirmed group, resulted in an area under the ROC curve (AUC) of 0.890 (Figure 2). With a cutoff value of 1.0, the sensitivity was 94.4% and the specificity was 57.6% (Table 1). Increasing the cutoff value results in an increase in specificity. Analysis limited to the normal adult group and the combined confirmed and probable groups resulted in an AUC of 0.794 with similar sensitivity and specificity trends.

**Table 1:** ROC analysis, sensitivity, and specificity of the Afmp1p antibody testing.

Groups	Area under curve (AUC)	Assay cutoff value	Sensitivity, %	Specificity, %
Normal adult vs. confirmed	0.890	1.0	94.4%	57.6%
		1.4	77.8%	87.9%
		1.7	50.0%	97.0%
Normal adult vs. confirmed and probable	0.794	1.0	83.9%	57.6%
		1.4	58.1%	87.9%
		1.7	32.3%	97.0%

### 3.2 Representative case examples

A 1.5-year-old male African penguin was part of a routine colony surveillance program for malaria, and opportunistic samples were submitted for analysis as part of the ongoing aspergillosis research study. The bird demonstrated no clinical signs during the entire study. Data from serological and electrophoresis testing are shown in Table 2, with the start of opportunistic sampling noted on day 0. On day 62, gliotoxin testing was positive and was concurrent with a marked decrease in the albumin:globulin (A/G) ratio relative to a decrease in albumin and increases in alpha 2 and beta globulins. On day 87, gliotoxin was no longer detected, but a clear seroconversion had occurred with a notable increase in Afmp1p antibody. The electrophoretogram continued to be abnormal but showed some improvements in albumin and beta globulins. On day 103, the electrophoretogram and a CBC (data not shown) were within normal limits with decreased antibody reactivity. This penguin continued to be clinically normal while at this facility for several months thereafter and was not administered any treatments. It was suspected that it had recovered from a subclinical *Aspergillus* infection during this time frame.

**Table 2:** Repeated measures in a 1.5-year-old male African penguin with presumed subclinical aspergillosis.

	Sampling date						
Test	Day 0	Day 62	Day 87	Day 103	Day 143	Day 178	Ref interval
Afmp1p (OD)	0.31	0.82	**1.99**	**1.42**	0.69	0.71	<1.0
Gliotoxin, ng/ml	<3.0	**>3.0**	<3.0	<3.0	<3.0	<3.0	<3.0^b^
Total Protein, g/dL	4.0	6.0	6.0	4.6	nd^a^	6.2	3.6-6.8
A/G ratio	1.22	**0.31**	**0.56**	1.18	nd^a^	0.99	0.79-1.60
Prealbumin, g/dL	0.31	0.22	0.37	0.21	nd^a^	0.37	0.23-0.90
Albumin, g/dL	1.88	**1.21**	1.78	2.28	nd^a^	2.62	1.43-2.87
Alpha 1, g/dL	0.06	0.07	0.11	0.10	nd^a^	0.19	0.06-0.22
Alpha 2, g/dL	0.43	**1.40**	**1.17**	0.70	nd^a^	1.13	0.31-1.05
Beta, g/dL	0.96	**2.35**	**1.89**	0.93	nd^a^	1.27	0.48-1.38
Gamma g/dL	0.36	0.76	0.69	0.38	nd^a^	0.43	0.18-0.95

Bold-faced results indicate abnormal/positive values.

^a^Not performed.

^b^Less than minimum detection level, <3 ng/mL.

An 8-year-old male African penguin presented with an occasional cough witnessed mostly at feeding time. A mild weight loss (0.5 kg, approximately 15%) was noted over the previous 7-month period. Radiographs revealed a right-sided opacity in the lungs and decreased volume, suggestive of pneumonia or neoplasia. Initial blood work included a 34% packed cell volume (PCV) and white blood cell (WBC) count of 16.4 × 10^3^/μl. Serological testing, gliotoxin detection, and protein electrophoresis data are shown in Table 3. A leukocytosis ranging from 20.4–63.0 × 10^3^/μl and a PCV of 25–34% was observed throughout the treatment period. The penguin was treated with anti-fungal medications and antibiotics through day 60. Anti-fungal treatment was discontinued but later re-initiated based on variations in clinical signs. A computed tomography (CT) scan on day 27 showed a marked improvement. Coughing abated on day 186 but was noted to return on day 246, at which point a decreased A/G ratio was noted. A follow-up CT on day 186 showed no residual pulmonary changes. One year after this period, clinical signs returned with the presence of gliotoxin, elevated Afmp1p antibody, and decreased A/G ratio. There was a positive response to antifungal treatment.

**Table 3:** Repeated measures in an 8-year-old male African penguin with confirmed clinical aspergillosis.

Sampling date										
Test	Day 0	Day 19	Day 48	Day 60	Day 83	Day 118	Day 186	Day 246	Day 301	Ref interval
Afmp1p, OD	**1.73**	**2.03**	**1.80**	**1.72**	**1.68**	**2.09**	**1.69**	**1.60**	**1.32**	<1.0
Gliotoxin, ng/ml	**18.0**	**63.0**	**43.0**	**12.8**	**21.0**	nd^a^	nd^a^	<3.0	<3.0	<3.0^b^
Hydroxybutyrate, mmol/L	0.65	0.51	0.56	0.55	0.32	**3.17**	nd^a^	0.70	0.56	0.19-1.94
Total Protein, g/dL	6.2	**7.2**	**8.0**	**7.8**	6.8	3.6	4.2	5.4	4.2	3.6–6.8
A/G ratio	**0.20**	**0.14**	**0.16**	**0.27**	**0.71**	0.91	1.40	**0.47**	1.41	0.79–1.60
Prealbumin, g/dL	**0.14**	**0.16**	**0.13**	**0.19**	0.21	0.42	0.24	0.20	0.22	0.23–0.90
Albumin, g/dL	**0.90**	**0.71**	**1.00**	1.45	2.60	1.30	2.21	1.52	2.23	1.43–2.87
Alpha 1, g/dL	0.11	0.15	0.13	0.08	0.07	0.04	0.07	0.12	0.09	0.06–0.22
Alpha 2, g/dL	**1.16**	**1.51**	**2.30**	**1.48**	**1.25**	0.48	0.45	1.04	0.47	0.31–1.05
Beta, g/dL	**2.64**	**3.16**	**2.79**	**2.98**	**1.63**	0.96	0.89	**1.87**	0.83	0.48–1.38
Gamma g/dL	**1.24**	**1.51**	**1.65**	**1.62**	**1.03**	0.40	0.34	0.65	0.35	0.18–0.95

Bold-faced results indicate abnormal/positive values.

^a^Not performed.

^B^less than minimum detection level, <3 ng/mL.

Eight additional cases of confirmed and probable aspergillosis are presented in Table 4. Note the consistent decrease in the A/G ratio in all cases. Seroconversion of the Afmp1p antibody occurred in all cases, with a noted borderline positive result (case 6). Gliotoxin was detected in all cases except for the Humboldt penguin (case 7).

**Table 4:** Test results from confirmed and probable cases of aspergillosis in different penguin species – Afmp1p antibody, gliotoxin, and A/G ratio derived from protein electrophoresis.

Case	Afmp1p (OD)	Gliotoxin ng/ml	A/G ratio	Case notes
1-African Penguin 8 yr. old female	**1.71**	**11.9**	**0.21**	Confirmed - 6-week course of treatment with worsening respiratory signs and inappetence; necropsy - severe aspergillosis with multifocal granulomas
2-Humboldt Penguin 20 years old female	**1.24**	**42.0**	**0.12**	Probable - Inappetence and lethargy, CT scan showed air sacculitis and plaques; resolution after 4 months of treatment
3-Humboldt Penguin 16 years old male	**2.23**	**27.0**	**0.23**	Confirmed – Inappetence, lethargy, and weight loss, CT showed severe luminal exudate; necropsy – severe aspergillosis
4-African Penguin 1.5 years old female	**2.64**	**>3.0**	**0.15**	Confirmed – Coughing, lethargy, and weight loss, 7-month course of treatment; necropsy – severe aspergillosis
5-Little Penguin 13 years old female	**2.64**	**>3.0**	**0.16**	Confirmed – Abnormal posture, lethargy, and dyspnea, radiographic evidence of mass in air sac and marked leukocytosis; necropsy – severe aspergillosis (*A. fumigatus*).
6-African Penguin 23 years old male	0.99	**>100**	**0.16**	Confirmed – 3-day course of treatment with acute signs and leukocytosis; necropsy - acute fulminant aspergillosis
7-Humboldt Penguin 13 years old male	**1.43**	<3.0^a^	**0.62**	Confirmed – 3-month course of treatment with weight loss, lethargy, hyporexia, and mass in right lung; anti-fungal and supportive treatment; necropsy – multicentric systemic *Aspergillus* infection; PCR – *Aspergillus* spp., not *A. fumigatus*
8-Gentoo Penguin 3 yr. old male	**1.50**	**3.9**	**0.37**	Probable – 4-month course of treatment with weight loss, persistent leukocytosis, abnormal vocalizations; improvement in electrophoresis concurrent with negative gliotoxin testing and decreased Afmp1p antibody with 45 days of anti-fungal treatment
Reference interval	**<1.0**	<3.0	**0.79–1.60**	

Bold-faced results indicate abnormal/positive values.

^a^Less than minimum detection level, <3 ng/ml.

Data presented from Case 1 is at the endpoint, approximately 5 days before euthanasia. The African penguin had previously been treated for aspergillosis 1 year earlier. The reemergence of infection was suspected with the presentation of a poor appetite. High levels of gliotoxin (62 ng/mL) were detected concurrently with a marked decrease in the A/G ratio and seropositive status. Supportive treatment and anti-fungal medications over 6 weeks resulted in a decrease in gliotoxin, but no change in A/G ratio or antibody reactivity was observed despite initial stabilization in clinical signs. Worsening respiratory signs and inappetence led to the decision of euthanasia. Aspergillosis was confirmed by necropsy.

Case 2, a case of probable aspergillosis in a Humboldt penguin based on CT, presented with inappetence and lethargy, at which point the initial sample (shown) was submitted for analysis. After 1 month of anti-fungal treatment, antibody reactivity increased to 2.14, gliotoxin was no longer detected, and the A/G ratio normalized to 0.89, with a decrease in alpha 2 and beta globulins. Afmp1p antibody reactivity decreased to 1.41 and 0.81 in follow-up testing at approximately 80- and 110-days post-presentation with consistent normal electrophoretograms and the absence of gliotoxin.

Case 4 presented with presumed aspergillosis, with coughing, lethargy, and weight loss. Initial CBC revealed leukocytosis (57.2 × 10^3^/μl), with an electrophoretogram with marked increases in alpha 2 and beta globulins. CT imaging confirmed multifocal pulmonary granulomas and air sacculitis. Treatment was concluded at month 2 with resolution of granulomas on imaging. The penguin remained strongly seropositive for Afmp1p reactivity (>2.0) throughout the following 6 months, during the period of recurrent clinical signs and repeat treatment (data shown in Table 4). The A/G ratio initially ranged from 0.54 to 0.73 but decreased to 0.15, at which point gliotoxin was also detected. Gliotoxin positive status persisted the following month in tandem with the markedly abnormal electrophoretogram. The penguin was euthanized 9 months after the original presentation, and aspergillosis was confirmed at necropsy.

Case 8, a gentoo penguin, was a probable case of aspergillosis with a presentation of significant weight loss and abnormal vocalizations. Initial blood work revealed mild leukocytosis with a marked decrease in the A/G ratio and Afmp1p antibody reactivity of 1.5 and a positive gliotoxin detection (data shown in Table 4). Over the next 30 days, gliotoxin peaked at 10.0 ng/mL, with Afmp1p antibody reactivity of 1.87. On day 83, the A/G ratio was 0.82 with normal levels of alpha 2 and beta globulins. This finding was concurrent with an improved physical exam and clinical signs, with Afmp1p antibody reactivity of 1.0, and no detection of gliotoxin.

Cases 3 and 5 through 7 were single-point assessments. Data from case 3 (Humboldt penguin) were obtained early after the onset of lethargy, weight loss, and inappetence. CT imaging supported the presence of infection, which was later supported by necropsy several months later. Case 5, a little blue penguin, was a confirmed case of aspergillosis based on clinical signs and radiographic data, which was later confirmed by necropsy as *A. fumigatus*. Case 6, an African penguin, had an acute fulminant infection with an initiation of treatment for only 3 days following the acute onset of clinical signs and leukocytosis. Notably, Afmp1p reactivity was borderline. Case 7 (Humboldt penguin) was a confirmed case of aspergillosis, although PCR testing and subsequent sequencing of tissues indicated a non-*A. fumigatus* species. This penguin initially presented with weight loss and lethargy, at which time this sample was obtained. Radiographs revealed a pulmonary mass. With anti-fungal treatment, a slight improvement was noted over a month, but clinical signs worsened 3.5 months after the initial presentation, at which time euthanasia was elected.

## 4 Discussion

In the present study, samples from various penguin species with probable and confirmed aspergillosis demonstrated antibody reactivity to Afmp1p, a secretory cell wall antigen of *Aspergillus* spp. By ROC analysis, a cutoff value of 1.0 provided very good sensitivity and moderate specificity. This differs from the value provided in the original report of the use of this assay in falcons, where a cutoff value of 0.4 was recommended (11). This may be due to the varying implementation of this assay as well as differences in immune response between falcons and penguins infected with *Aspergillus* spp. The low falcon cutoff value is similar to that observed in the normal juvenile penguin group (median 0.56), which may also indicate a lower level of exposure to *Aspergillus* spp. and/or possible low-level infection. In addition, the use of different anti-IgY conjugate antibodies will affect the results. In the case of the falcon study, a polyclonal anti-falcon antibody was prepared, whereas in the present study, a cross-reactive anti-chicken antibody was utilized. Polyclonal antibody preparations may vary in cross-reactivity across avian species and may also vary in reactivity by lot number; thus, care should be taken when this type of assay is implemented.

Interestingly, higher values of Afmp1p antibody reactivity were found in a group of clinically normal adult African penguins (median age 9 years) compared to a clinically normal group of juvenile African penguins (median age 1.7 years). While these two groups of African penguins were obtained from two facilities, which likely have differences in husbandry, housing, and possible exposure to *Aspergillus* spp., it could also be proposed that penguins will be exposed to this ubiquitous fungal agent multiple times and that a higher “normal” level of antibody may be acquired over a lifetime. Even within the normal adult group, the reactivity of 0.72–1.95 demonstrated this potential wide variance in antibody levels. While the normal adult data set was limited (*n* = 33), it may be notable that Afmp1p antibody reactivity was significantly correlated with age. Additional studies should be undertaken to examine this association in other penguin species and across facilities, perhaps by season if outdoor housing is present. Conceptually, this increased reactivity may reflect the high levels of antibody reactivity when using the original whole antigen ELISA, which rendered the latter assay not applicable for use in penguins (4, 6). It may also be proposed that the knowledge of antibody reactivity by facility or by individual penguin may be helpful, as it would provide a baseline antibody reactivity that could be compared to laboratory results from the same individual in the event of suspected fungal infection. Future studies should focus on the benefits of annual health assessment testing or, at a minimum, annual banking of samples, which would be available for comparison if a penguin begins to show clinical signs.

The case of the juvenile African penguin (Table 2) may offer some insights as to the timeline of the immune response to infection, as well as reflect differences by age. The samples from juvenile penguins were acquired as part of a routine monitoring program for avian malaria, which were banked for use in the current study. The veterinary staff at this facility (co-author EB) reports that reactive lymphocytes are commonly observed in the spring and summer seasons in juvenile penguins and hypothesize that this is reflective of their first exposure to *Aspergillus,* which is correlated to increased fungal loads detected by environmental monitoring of outdoor areas (13, 14). Notably, the penguin profiled in Table 2 remained clinically normal throughout and after the completion of the study. The first indication of a subclinical infection was the concurrent detection of gliotoxin with typical electrophoretic changes in globulin fractions. This was followed by Afmp1p antibody seroconversion, during which the A/G ratio was starting to recover and gliotoxin was no longer detectable. While the sampling timepoints were not stringent, these data reflect the initial acute phase response, which was followed by the cell-mediated immune response. Notably, no anti-fungal treatment was given in this case; the immune response of this penguin was rigorous enough to not lead to the development of any clinical signs and not have any lasting complications. It is noteworthy that later sampling of this penguin returned antibody reactivity levels approaching the mean value of the normal juvenile penguin group. This contrasts with other cases in adult penguins where, despite apparent successful treatment, antibody reactivity may decrease but never approximate juvenile penguin levels.

Understanding the relative utility of any one assay, including Afmp1p antibody testing for prognosis, is difficult as the current study does not have a large sample set of complete repeated measures of penguins that responded to treatment for aspergillosis. In one case, downward trending antibody reactivity was observed concurrent with the decreased gliotoxin, supporting a positive response to treatment. In another case, antibody levels initially increased in the presence of continued gliotoxin and then later decreased while positive changes in the electrophoretogram began to steadily normalize. At the present time, resolution of a decreased A/G ratio is the most consistent marker of a return to health.

In the challenging cases that led to euthanasia or death, levels of Afmp1p antibody reactivity persisted in moderate to high ranges. In case 1 (Table 4), antibody levels ranged from 1.67 to 1.96 over the treatment period with a wide range of gliotoxin (11–80 ng/mL) and a persistently low A/G ratio (0.10–0.21). The albumin ranged from 0.43 to 1.15 g/dL. Hypoalbuminemia and poor case outcome are consistent with those previously reported in gentoo penguins (15). In case 4, anti-fungal treatment was pursued for several months, where antibody levels were consistently greater than 2.0. The A/G ratio improved over the first 4 months but thereafter decreased with a worsening A/G ratio, and the detection of gliotoxin is consistent with the progression of infection. Case 6 appeared to be an acute fulminant infection with no seroconversion, which may be related to the brief time period of infection.

The other cases presented in Table 4 illustrate that a single test approach may not be beneficial. While each case sample did reflect a decreased A/G ratio, the antibody reactivity varied from 0.99 (borderline negative) to 2.23, with most reflecting moderate increases from 1.2 to 1.5. The lack of a more robust antibody response may reflect the timing of the immune response at the sampling date or perhaps the severity of the infection at that time. In 7 out of 8 cases, gliotoxin was detected. It was not detected in case 7, the penguin was infected with a non-*fumigatus Aspergillus* species, which is consistent with reports that gliotoxin is primarily made by *A. fumigatus*, the more common species of *Aspergillus* in penguin cases (16, 17). This test is otherwise considered highly specific but the sensitivity is poorer in birds with probable infection (10). This may be a bias in the samples selected for the study, but it may indicate that gliotoxin is more likely to be present in advanced infection. As gliotoxin is considered immune suppressive, it may be proposed to account for some of the variability in the antibody levels (18, 19). In total, these cases demonstrate that the use of the panel approach (Afmp1p antibody, gliotoxin, electrophoresis) may be most beneficial, at least in early diagnostic testing of suspected cases, with a follow-up testing of gliotoxin (if initially positive) and electrophoresis for prognostic information. Afmp1p antibody reactivity can be helpful if the results are very elevated and may also be applicable in routine health screening of younger penguins. In adults, baseline reactivity may need to be determined or examined through the use of repeated measures for increasing reactivity in penguins with suspected aspergillosis. These tests can be used to complement information gained by CBC and other routine clinical measures. This is consistent with a recent systematic review that no single test for avian aspergillosis can provide definitive diagnostic information in all case presentations (2).

A previous study examined the application of a commercially available Western blot for the detection of antibodies to *Aspergillus* in various penguin species (20). While a variability in results was observed, likely due to facility differences and limited sample size, this method did provide a novel view of antibody reactivity of normal penguins as well as penguins with confirmed infection. While there was an overlap with particular *Aspergillus* antigens that were also the focus of antibody responses in infected humans, there were some antigens that appeared to be unique. Future studies may benefit from producing a wider panel of *Aspergillus* antigens for antibody reactivity testing with the aim of distinguishing exposure from infection. Based on the current results, continued examination of Afmp1p reactivity in penguins and other avian species is warranted.

## Data Availability

The raw data supporting the conclusions of this article will be made available by the authors, without undue reservation.

## References

[1] Fischer D Lierz M. Diagnostic procedures and available techniques for the diagnosis of aspergillosis in birds. J Exot Pet Med. (2015) 24:283–95. doi: 10.1053/j.jepm.2015.06.016

[2] Vieu S Guillot J Beaudeau F. Antemortem diagnostic tests for the detection of *aspergillus* infection in birds: a systematic review. Med Mycol. (2024) 62. doi: 10.1093/mmy/myae112, PMID: 39544133

[3] Arné P Risco-Castillo V Jouvion G Le Barzic C Guillot J. Aspergillosis in wild birds. J Fungi. (2021) 7:241. doi: 10.3390/jof7030241, PMID: 33807065 PMC8004873

[4] Desoubeaux G Rodriguez M Bronson E Sirpenski G Cray C. Application of 3-hydroxybutyrate measurement and plasma protein electrophoresis in the diagnosis of aspergillosis in African penguins (*Spheniscus demersus*). J Zoo Wildl Med. (2018) 49:696–703. doi: 10.1638/2017-0172.1, PMID: 30212328

[5] Hauck R Cray C França M. Spotlight on avian pathology: aspergillosis. Avian Pathol. (2020) 49:115–8. doi: 10.1080/03079457.2019.1696946, PMID: 31766868

[6] Cray C Watson T Arheart KL. Serosurvey and diagnostic application of antibody titers to *aspergillus* in avian species. Avian Dis. (2009) 53:491–4. doi: 10.1637/8673-030209-Reg.1, PMID: 20095147

[7] Fischer D Van Waeyenberghe L Cray C Gross M Usleber E Pasmans F. Comparison of diagnostic tools for the detection of aspergillosis in blood samples of experimentally infected falcons. Av Dis. (2014) 58:587–98. doi: 10.1637/10831-032714-Reg, PMID: 25619004

[8] Cray C Reavill DR Romagnano A Van Sant F Champagne D Stevenson R. Galactomannan assay and protein electrophoresis findings in psittacine birds with aspergillosis. J Av Med Surg. (2009) 23:125–35. doi: 10.1647/2007-041.1, PMID: 19673459

[9] Cray C Watson T Rodriguez M Arheart KL. Application of galactomannan analysis and protein electrophoresis in the diagnosis of aspergillosis in avian species. J Zoo Wildl Med. (2009) 40:64–70. doi: 10.1638/2007-0138.1, PMID: 19368241

[10] Reidy L Desoubeaux G Cardenas J Seither J Kahl K Chauvin D. Detection of gliotoxin but not bis(methyl)gliotoxin in plasma from birds with confirmed and probable aspergillosis. J Zoo Wildl Med. (2022) 53:10:60–9. doi: 10.1638/2021-0070 35339150

[11] Wernery U Tsang C-C Hebel C Damerau A Kinne J Cai J-P. Serodiagnosis of aspergillosis in falcons (*Falco* spp.) by an Afmp1p-based enzyme-linked immunosorbent assay. Mycoses. (2018) 61:600–9. doi: 10.1111/myc.12776, PMID: 29611232

[12] Yuen KY Chan CM Chan KM Woo PC Che XY Leung AS. Characterization of AFMP1: a novel target for serodiagnosis of aspergillosis. J Clin Microbiol. (2001) 39:3830–7. doi: 10.1128/JCM.39.11.3830-3837.2001, PMID: 11682494 PMC88451

[13] Cateau E Leclerc A Cartier N Valsecchi I Bailly É Le Senechal R. Aspergillosis in a colony of Humboldt penguins (*Spheniscus humboldti*) under managed care: a clinical and environmental investigation in a French zoological park. Med Mycol. (2022) 60. doi: 10.1093/mmy/myac046 35713494

[14] Rivas AE Dykstra MJ Kranz K Bronson E. Environmental fungal loads in an indoor–outdoor African penguin (*Spheniscus demersus*) exhibit. J Zoo Wildl Med. (2018) 49:542–55. doi: 10.1638/2017-0119.1, PMID: 30212323

[15] Naylor AD Girling SJ Brown D Crompton CG Pizzi R. Plasma protein electrophoresis as a prognostic indicator in *aspergillus* species-infected Gentoo penguins (*Pygoscelis papua papua*). Vet Clin Pathol. (2017) 46:605–14. doi: 10.1111/vcp.12527 28692132

[16] Kupfahl C Michalka A Lass-Florl C Fischer G Haase G Ruppert T. Gliotoxin production by clinical and environmental *Aspergillus fumigatus* strains. Intl J Med Microbiol. (2008) 298:319–27. doi: 10.1016/j.ijmm.2007.04.006, PMID: 17574915

[17] Lewis RE Wiederhold NP Lionakis MS Prince RA Kontoyiannis DP. Frequency and species distribution of gliotoxin-producing *aspergillus* isolates recovered from patients at a tertiary-care cancer center. J Clin Microbiol. (2005) 43:6120–2. doi: 10.1128/JCM.43.12.6120-6122.2005, PMID: 16333108 PMC1317213

[18] Arias M Santiago L Vidal-Garcia M Redrado S Lanuza P Comas L. Preparations for invasion: modulation of host lung immunity during pulmonary aspergillosis by gliotoxin and other fungal secondary metabolites. Front Immunol. (2018) 9:2549. doi: 10.3389/fimmu.2018.02549, PMID: 30459771 PMC6232612

[19] Kwon-Chung KJ Sugui JA. What do we know about the role of gliotoxin in the pathobiology of *Aspergillus fumigatus*? Med Mycol. (2009) 47:S97–S103. doi: 10.1080/13693780802056012, PMID: 18608908 PMC2729542

[20] Leclerc A Piarroux R Callico A Bronson E Cray C. Adaptation of a commercially available Western blot kit for the detection of antibody to A*spergillus* in penguins in France and the United States. J Zoo Wildl Med. (2024) 55:595–601. doi: 10.1638/2024-0008, PMID: 39255200

